# Parsing interindividual drug variability: an emerging role for systems pharmacology

**DOI:** 10.1002/wsbm.1302

**Published:** 2015-05-07

**Authors:** Richard M Turner, B Kevin Park, Munir Pirmohamed

**Affiliations:** 1 The Wolfson Centre for Personalised Medicine, Institute for Translational Medicine, University of LiverpoolLiverpool, UK; 2 MRC Centre for Drug Safety Science, Department of Molecular and Clinical Pharmacology, University of LiverpoolLiverpool, UK

## Abstract

There is notable interindividual heterogeneity in drug response, affecting both drug efficacy and toxicity, resulting in patient harm and the inefficient utilization of limited healthcare resources. Pharmacogenomics is at the forefront of research to understand interindividual drug response variability, but although many genotype-drug response associations have been identified, translation of pharmacogenomic associations into clinical practice has been hampered by inconsistent findings and inadequate predictive values. These limitations are in part due to the complex interplay between drug-specific, human body and environmental factors influencing drug response and therefore pharmacogenomics, whilst intrinsically necessary, is by itself unlikely to adequately parse drug variability. The emergent, interdisciplinary and rapidly developing field of systems pharmacology, which incorporates but goes beyond pharmacogenomics, holds significant potential to further parse interindividual drug variability. Systems pharmacology broadly encompasses two distinct research efforts, pharmacologically-orientated systems biology and pharmacometrics. Pharmacologically-orientated systems biology utilizes high throughput omics technologies, including next-generation sequencing, transcriptomics and proteomics, to identify factors associated with differential drug response within the different levels of biological organization in the hierarchical human body. Increasingly complex pharmacometric models are being developed that quantitatively integrate factors associated with drug response. Although distinct, these research areas complement one another and continual development can be facilitated by iterating between dynamic experimental and computational findings. Ultimately, quantitative data-derived models of sufficient detail will be required to help realize the goal of precision medicine. *WIREs Syst Biol Med* 2015, 7:221–241. doi: 10.1002/wsbm.1302

## INTRODUCTION

Drug treatment is the most common therapeutic intervention advocated for patients by physicians, and its prevalence is increasing. By 2018, it is forecast that global annual spending on prescription medications will reach $1.3 trillion.^1^ However, there exists notable interindividual heterogeneity in drug response, affecting both efficacy and toxicity. It is has been reported that the proportion of patients who respond beneficially to the first drug offered in the treatment of a wide range of diseases is typically just 50–75%.^2^ Approximately 6.5% of admissions to hospitals are related to adverse drug reactions (ADRs)^3^ and about 15% of inpatients experience an ADR.^4^ Therefore collectively, interindividual drug variability leads to patient harm and the excessive and inefficient use of limited healthcare resources.

Pharmacogenomics is the study of genetic determinants of interindividual variation in response to a given drug and was developed to optimize drug use through the stratification of pharmacological therapy by patient subgroup, via genotype-informed drug and dose selection. Currently, 138 US Food and Drug Administration (FDA)-approved drugs have at least one pharmacogenomic association in their product labelling.^5^ Pharmacogenomics has been successfully translated into clinical practice, notably:

1. reducing the hypersensitivity syndrome associated with the antiretroviral abacavir, through genotyping for HLA-B*57:01, and
2. the development of oncogenotype-specific drugs, such as the *V600E*-specific BRAF inhibitor vemurafenib.

However, the hype following completion of the Human Genome Project of a paradigm shift into an era of pharmacogenomics-mediated precision medicine has not occurred. There are many reasons for this (covered in other review articles^6^,^7^), but it is also important to appreciate that most forms of drug response are, in effect, complex phenotypes (like complex diseases). Thus, pharmacogenomics combined with traditional clinical phenotypic variables (e.g., sex and age) alone are unlikely sufficient to adequately describe the net effect of the multitude of factors influencing drug response. Therefore, there is growing interest in the novel, diverse, and rapid developing field of systems pharmacology, which incorporates but goes beyond pharmacogenomics, to help realize the goal of precision medicine. This review is subdivided into three sections: an overview of systems pharmacology, a necessary step back to systematically consider the factors affecting drug response and finally an overview of systems pharmacology approaches in practice to help parse interindividual drug response variability.

## AN OVERVIEW OF SYSTEMS PHARMACOLOGY

The conventional reductionist approach to understanding drug action and drug design has been the *one disease, one gene, one drug* paradigm, attributing drug effectiveness to its action on a single on-target protein within the target tissue. This analogy has been extended to account for the plethora of ADRs caused by a drug by dividing them into excessive on-target effects, on-target effects in nontargeted tissues, and off-target drug activity. However, for the pharmaceutical industry it has become increasingly clear that *business as usual is not an option*.^8^ It costs approximately one billion USD to bring a single drug to market and despite the considerably increased pharmaceutical R&D investment this century, the number of new molecular entities approved annually by the FDA has remained constant at ∼20–30 compounds.^9^ The highest attrition rate during the clinical phases of drug development now occurs in phase II trials where the development of 75% of investigational medicinal products is terminated, largely due to poor drug effectiveness and unanticipated and/or unacceptable toxicity.^9^ There is a growing appreciation that the reductionist approach to molecular-based drug design is failing because it insufficiently considers both the complexity of the interactions between a drug and the human body and the complexity of the body's response to drug perturbation. This lack of understanding manifests as unexpected ineffectiveness and ADRs. This also contrasts with drug–drug interactions that have a pharmacokinetic (PK) basis, where knowledge of drug metabolizing enzymes (DMEs) and xenobiotic transporters (XTs) has led to the development of *in vitro*, *in silico*, and *in vivo* methods that can be used to predict the possibility of drug interactions in clinical practice.

Systems pharmacology is an emergent field of interdisciplinary translational science,^10^ and therefore has no single consensus definition, instilling divergent connotative meanings between researchers. Overall, systems pharmacology is a holistic approach to pharmacology that aims to systematically and comprehensively parse all of a drug's clinically relevant activities in the human body to explain, simulate and predict the resultant clinical drug response. It is hoped that systems pharmacology will accelerate drug discovery and development through identifying and validating new targets, understanding target network responses to drug perturbation and uncovering drug-response biomarkers.^10^ However, the application of systems pharmacology holds additional transformative potential for a deeper parsing of interindividual drug variability of existing and new drugs, facilitating drug stratification.

**FIGURE 1:**
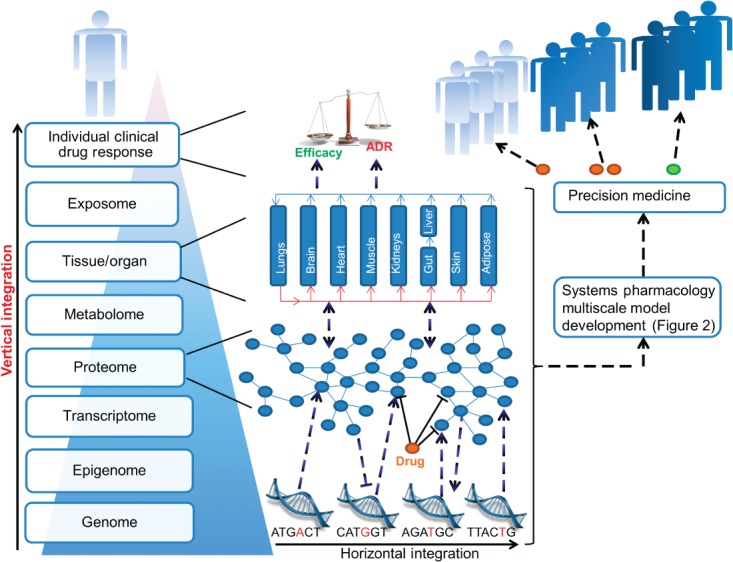
The rationale for a multiscale network-based understanding of drug action. The human body can be parsed into a hierarchy of biological levels; interactions within and between levels form networks that are interconnected to other networks, resulting in the complex human body system. The founder constituents of this dynamic complex system are the genome and exposome; the latter represents environmental exposures (e.g., smoking) that interact with and influence all biological levels of the human body. Many drugs have more than one protein target and therefore a network-based understanding is more informative than a single target perspective. In this figure, the genomic, proteomic and tissue/organ levels have been expanded, although all levels can inform an individual's response to drug therapy. Genetic polymorphisms can, e.g., alter the structure and/or abundance of a drug target and important proteins mediating the drug-induced proteomic network response. This network response influences other levels in different times and spaces, for example altering gene transcription and tissue function. Intra- and inter-level interactions ultimately lead to the emergence of an individual patient's clinical drug response. Through investigating and modelling these interactions using empirical and pharmacometric methods, illustrated further in Figure 2, the aim is to develop multiscale models to facilitate dose- and drug-adjusted precision medicine. ADR: adverse drug reaction

It is evident that the human body is greater than the sum of its parts; clinical drug response is one emergent property of this synergy, which plausibly stems from the body's hierarchical, network-based design. Humans are composed of different scales (i.e., levels) of biological organization acting in different times and spaces: molecular, genomic, epigenomic, transcriptomic, proteomic, metabolomic, cell, gut microbiome, tissue, organ, and whole body. Within and between levels, molecules are interlinked to form biological networks (e.g., macromolecular protein–protein structures and gene regulatory networks).^9^ Therefore understanding drug action is intrinsically a multiscale,^10^ network-based problem (Figure 1) and so rather than seeking to understand the effects of drugs through the *ad hoc* study of individual biological components, systems pharmacology aims to understand drug-induced network perturbations as a whole through the investigation and integration of:

1. the effects of a given drug within different biological levels (horizontal integration) and
2. drug-induced interactions between different biological levels (vertical integration).

From this multiscale context incorporating biological network properties, such as robustness and redundancy,^9^ it is clear why, for example, *in vitro* drug binding to a single target molecule cannot readily be extrapolated to beneficial clinical response with no unexpected harm.

**FIGURE 2:**
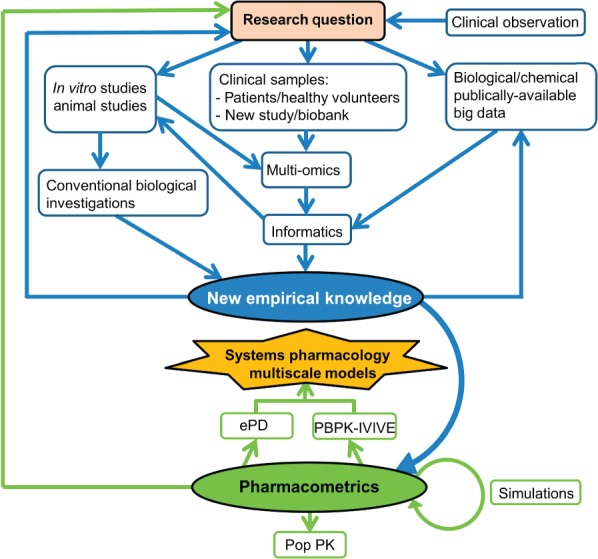
The interrelated processes for systems pharmacology multiscale model development. This figure provides a nonexhaustive overview of the processes and interconnections relevant to multiscale modelling. First, from a clinical observation and/or new research finding, a new research question is generated. Three major empirical resources can be harnessed to address the question: clinical, *in vitro*/animal and publically available empirically derived databases. Multi-omics approaches coupled with bioinformatics can uncover new associations. Network description and analysis can glean further information from existing databases (e.g., of high throughput data), predicting new targets and defining molecular sub-networks associated with drug response phenotypes of interest (e.g., adverse drug reactions). Conventional biological investigations can validate these new associations and predictions, derive mechanistic insight, and perform detailed biochemical kinetics analyses. This empirical data can be incorporated into quantitative pharmacometric models. Population pharmacokinetics (POP PK) top down modelling is tightly fitted to empirical data. However physiologically based PK (PBPK) coupled with *in vitro–in vivo* extrapolation (IVIVE) and enhanced pharmacodynamics (ePD) modelling are more bottom up, using empirical data where available and assumptions when necessary. Model simulations and assumptions will drive further empirical experimentation, leading to an iterative process of model development and refinement. Through combining detailed PBPK-IVIVE and ePD models that are adequately fitted to empirical data, systems pharmacology multiscale models with adequate predictive power to facilitate precision medicine will hopefully be developed.

Systems pharmacology is composed of, and the interface between, two broad-based but complementary research themes: the application of systems biology knowledge and skills to study system-wide drug effects, and pharmacometrics.^11^ Interest in systems biology has grown dramatically in recent years for several reasons including increasing experimental omics technology and network analysis capabilities, and the growing involvement of systems engineers, mathematicians, computer scientists, and physicists in addressing biological problems.^10^ Pharmacology is fundamentally a quantitative science and pharmacometrics aims to develop quantitative mathematical models to describe a drug's PK and pharmacodynamic (PD) properties, quantify uncertainty and rationalize data-driven decision making in drug development and pharmacotherapy.^12^ Given the two extremes of firstly, the indiscernible complexity resulting from summation of all potential biological interactions in the human body to secondly, the reductionist overly simplistic target-level only understanding of drug action, systems pharmacology aspires to compromise and develop contemporary models in the short to medium term that are both achievable and sufficiently informative to adequately describe drug variability for clinical utility. It is envisaged that high throughput omics technologies and network-based analyses, alongside conventional experimental techniques, will identify novel factors from multiple different biological levels associated with different clinical drug response phenotypes. Mechanistic and kinetics analyses of these factors will facilitate their quantitative integration, leading to the construction of multiscale systems pharmacology models. The model development process is iterative as new empirical knowledge refines the model and model simulations drive new empirical studies^10^ (Figure 2).

## FACTORS ASSOCIATED WITH INTERINDIVIDUAL DRUG RESPONSE

Before considering systems pharmacology approaches for parsing interindividual drug response variability in more detail, it is informative to first take a step back and review the categories of factors shaping drug response. Classically, pharmacology is delineated into PK (‘*what the body does to the drug*’) and PD (‘*what the drug does to the body*’) and PK is further subdivided into ADME constituents: absorption, distribution, metabolism, and excretion. As drug availability (PK) at its target site(s) is a prerequisite for drug action (PD), both PK and PD contribute to drug response. For any given drug, both PK and PD are the products of the characteristics and interactions between the drug and the overall human body system. The major system inputs are the genome and myriad environmental exposures (past and present), which shape each other and shape the dynamic biological levels (e.g., epigenome, transcriptome, proteome, metabolome, and tissues/organs); in turn these levels interact with one another through complex processes. Therefore, within each biological level of the human body, factors associated with drug response exist. Drug-specific, human body and environmental factors are now each sequentially reviewed (summarized in Table 1,^13^–^45^). Given the interconnectedness between human body constituents and environmental factors, their separation represents a pragmatic simplification. Nevertheless, this framework is inclusive and conceptually useful. The role of analytical variation in determining PK/PD, although important, is not considered further here.

**TABLE 1:** Examples of Drug, Human Body Level, and Environmental Factors Associated with Interindividual Drug Response Variability

	Example		
Factor Classification	Factor	Effect	Reference
*Drug-specific*			
Drug-delivery	Diltiazem single versus dual microbead oral delivery	↑ AUC + *C*_max_ with single microbead	Ref. 13
Drug regimen	Variable adherence to medication (e.g., long-term statin therapy)	↑ risk of cardiovascular events with reduced statin adherence	Ref. 14
*Human body levels*			
Genome			
On-target	Tumour Bcr-Abl T315I	↑ imatinib resistance	Ref. 15
	*VKORC1* rs9923231	↓ WSD requirements	Ref. 16
	*CYP4F2* rs2108622^1^	↑ WSD requirements	Refs 17–18
Off-target	HLA-B*57:01	↑ risk of abacavir hypersensitivity syndrome	Refs 19–20
DME function	CYP2C9*2, *3	↓ WSD requirements; ↑ risk of haemorrhage (CYP2C9*2,*3 and VKORC1 rs9923231 combined)	Refs 16, 21
	CYP3A5*3	↓ tacrolimus dose requirements	Ref. 22
	CYP3A4*22	↓ tacrolimus dose requirements	Ref. 23
	POR*28^2^	↓ dose-adjusted tacrolimus trough levels	Ref. 24
XTs	*SLCO1B1* rs4149056	↑ risk of simvastatin-induced myopathy; ↑ plasma exposure of several statins including simvastatin acid + rosuvastatin	Refs 25–27
	*ABCG2* rs4148162	↓ absorption of simvastatin	Ref. 25
Transcription factors	*PPARA* rs4253728 or rs4823613^2^	↑ tacrolimus dose-adjusted trough blood concentration	Ref. 28
Epigenome			
DNA methylation	*CYP3A4* methylation near proximal promoter	Associated with CYP3A4 mRNA expression	Ref. 29
	*TFAP2E* hypermethylation	Clinical nonresponse to 5-FU in colorectal cancer	Ref. 30
	*MGMT* promoter methylation	↑ overall survival with PCV chemotherapy in gliomas	Ref. 31
MicroRNA	↑ miR-519a expression	↓ tamoxifen-induced apoptosis; ↓ disease-free survival in oestrogen receptor positive breast cancer	Ref. 32
Proteome			
Protein synthesis regulation	↑ inflammatory cytokines (e.g., IL-6)	↓ transcription of hepatic DMEs/XTs	Ref. 33
Serum protein levels	Multivariate protein test based on eight serum mass spectra intensity levels	Associated with overall survival outcomes with erlotinib	Ref. 34
Metabolome			
Purine metabolism	Inosine and adenosine levels	↑ in aspirin poor responders	Ref. 35
Tissue/organ			
Anatomical	↑ adiposity	↑ distribution of lipophilic drugs into fatty tissue	
	Small liver/kidney (e.g., infant)	↓ drug metabolism/excretion	
Physiological	↑ intestinal transit time	↓ predicted oral absorption of poorly soluble/low permeability/slow release drugs	Ref. 36
Pathophysiological	Clinical liver or kidney disease	↓ drug metabolism/excretion	
	Altered blood flow	Precedes hepatocellular injury in paracetamol hepatotoxicity	Ref. 37
Iatrogenic	Gastrectomy	↑ AUC + *C*_max_ of 5-FU after S-1 ingestion	Ref. 38
*Environmental*			
Drugs, smoking, and concomitant food intake	PD interaction	Verapamil/beta blocker interaction to ↑ bradycardia/hypotension	
	Absorption	↓ levothyroxine absorption by e.g., iron, calcium, sevelamer	Ref. 39
	DME and XT inhibition	Grapefruit juice inhibits intestinal CYP3A4; Saquinavir/ritonavir inhibit CYP3A4 and OATP1B1 leading to ↑ simvastatin acid AUC + *C*_max_	Refs 40–41
	DME and XT induction	↑ CYP3A4/3A5 by carbamazepine; ↑ CYP2B6, 2C8, 2C9, 2C19, 3A4/3A5, ABCB1, and specific phase II enzymes (e.g., UGT1A1) with chronic rifampicin; ↑ CYP1A1 and 1A2 by smoking	Refs 42–44
UV exposure	CYP3A4 induction	Seasonal variation in duodenal CYP3A4 mRNA; Seasonal trend for variation in midazolam AUC	Ref. 45

AUC, area under the plasma drug concentration time curve; *C*_max_, maximum plasma concentration; DME, drug-metabolizing enzyme; 5-FU, 5-fluorouracil; IL-6, Interleukin-6; PCV, procarbazine, CCNU and vincristine adjuvant chemotherapy regimen; S-1, composite drug of 5-FU, tegafur, and two 5-FU modulatory compounds; WSD, warfarin stable dose; XT, xenobiotic transporter.

1 CYP4F2 does do not directly interact with warfarin but rs2108622 indirectly affects warfarin pharmacodynamics.

2 Gene protein products do not directly interact with specified drug but genetic variants indirectly perturb drug pharmacokinetics.

### Drug-Specific Factors

Two categories of drug-specific factors affect drug response:

1. the physicochemical properties of the drug and
2. the drug regimen.

Different drugs have different physicochemical properties, such as solubility, permeability, and propensity for interaction with a given available biological molecule (based on both three-dimensional shape and chemical/electrostatic forces complementarity^15^); these properties are the foundation of the distinct action of different drugs. However, drug-delivery modification (e.g., by varying the formulation or delivery system) is an important method to manipulate the properties of a given drug, alter its absorption profile and influence response. For example, using the same 240 mg once daily oral dose of the calcium channel antagonist, diltiazem, a single microbead delivery system has been shown to increase the area under the plasma concentration–time curve (AUC), increase the maximum plasma concentration (*C*_max_), and significantly lower ambulatory blood pressure in hypertensive patients, compared to a dual bead system.^13^

Secondly, drug regimen (dose, administration frequency, timing, and route) shapes PK. For example, patients with diabetes mellitus requiring insulin can personalize the dose, frequency, and timing of insulin therapy based on blood glucose readings, in conjunction with selection of the appropriate insulin formulation(s) for their insulin regimen. This is important as intensive blood glucose control is associated with a decreased risk of microvascular complications (nephropathy, neuropathy, and retinopathy).^46^

Importantly patient nonadherence is a substantial contributor to the variability in drug response observed in clinical practice. For example, in a study of asthma medication adherence, 35 and 45% of patients filled just ≤50 and 51–100% of inhaled medication prescriptions, respectively.^47^ However, the factors (including behavioral) underlying variable adherence are poorly understood. Interestingly, a recent U.S. internet survey that investigated statin use reported that the most frequent reason for statin discontinuation was statin-associated muscle adverse symptoms^48^ and current statin users who reported muscle symptoms were less likely to be adherent with statin theapy.^48^ This illustrates the potential contribution of even mild ADRs to variable patient adherence and importantly, statin nonadherence is associated with an increased risk of cardiovascular events.^14^

### Human Body Factors

At each level of biological organization within the human body, factors influence drug PK and PD. Heuristically these factors affect either the intrinsic *quality* (e.g., affinity and catalytic capacity) of the drug-biological molecule (predominantly protein) interaction or the *quantity* of molecule available for a drug to interact with (e.g., via synthesis and degradation). Quality and quantity considerations can also be extended to biological molecule–biological molecule interactions (e.g., protein–DNA, protein–protein, and microRNA–mRNA) that shape the network-level response to drug perturbation.

In the following sections, factors at the genomic, epigenomic, proteomic, and tissue/organ levels that affect a drug's PK or PD are considered, although other biological levels including the metabolome and gut microbiome are also of emerging importance.

#### Pharmacogenomics of Drug Response

The following drug-centric examples collectively illustrate the effects of pharmacogenomics variants on: drug PK and on/off-target PD, interaction quality and protein quantity, and the variable translational success of pharmacogenomics.

##### Warfarin-Dose Requirements

The vitamin K antagonist warfarin is a coumarin-derived racemic anticoagulant that has a narrow therapeutic index: the risks of haemorrhage and thromboembolism depend on the international normalized ratio (INR). The genetic variants rs9923231 (−1639G > A; G3673A) in *VKORC1*, CYP2C9*2 (rs1799853, R144C), CYP2C9*3 (rs1057910, I359L), and rs2108622 (1297G > A, V433M) in *CYP4F2* are established pharmacogenomic factors associated with warfarin stable dose (WSD) requirements,^16^,^17^ although their impact is ethnicity-dependent.^7^

rs9923231 is a noncoding single nucleotide polymorphism (SNP) that perturbs a transcription binding site in the promoter region of *VKORC1*, which encodes for the warfarin target, vitamin K epoxide reductase complex subunit 1^49^; rs9923231 decreases *VKORC1* expression and is associated with decreased WSD requirements.^16^ Cytochrome P450 2C9 (CYP2C9) metabolically inactivates the more potent *S*-warfarin enantiomer. CYP2C9*2 and CYP2C9*3 are missense reduction-of-function variants that decrease enzymatic activity by ∼30–40 and ∼80–90%, respectively,^50^ are associated with prolonged warfarin half-life and reduced WSD requirements.^16^ Genome-wide studies identified that rs2108622 in *CYP4F2* is associated with increased WSD requirements in Caucasian^17^ and Asian populations,^18^ but not in African-Americans.^51^ CYP4F2 depletes the vitamin K cycle of active vitamin K; rs2108622 is associated with lower hepatic CYP4F2 concentrations, greater vitamin K availability^52^ and illustrates the functional relevance of variation beyond proteins that directly interact with a drug.

Two recent large prospective warfarin pharmacogenomics randomized controlled trials (RCTs) that tailored warfarin therapy based on genotype information, COAG^53^ and EU-PACT,^54^ have produced contrasting results on the utility of empirically derived pharmacogenomic-based (*VKORC1* −1639G > A, CYP2C9*2 and *3) warfarin dosing algorithms, illustrating the difficulties encountered when seeking to translate even robust pharmacogenomic associations. Whilst EU-PACT reported a significantly improved time within INR therapeutic range (TTR) over 12 weeks from starting warfarin in the pharmacogenomic arm,^54^ COAG found no difference in TTR over the initial four weeks, compared to their respective control arms.^53^ There are various potential reasons for the contrasting results between these two trials, including differences in algorithmic strategy, the differences in ethnic heterogeneity between the two trials, and how the genotypes were utilized prior to the initiation of warfarin dosing.^55^ Other trials such as the ongoing GIFT RCT may provide further clarity in the future, but will also critically be dependent on the algorithms used and how non-Caucasian populations are dosed.

Importantly, a large prespecified genetic analysis of a multicentre RCT, which compared warfarin to the direct factor Xa inhibitor edoxaban in patients with nonvalvular atrial fibrillation, has highlighted the impact of genetic factors in contributing to clinical outcomes from warfarin therapy.^21^ Patients who were sensitive or highly sensitive to warfarin, based on combined *VKORC1* rs9923231 and CYP2C9*2/*3 genotype status, spent greater proportions of time over-anticoagulated and had an increased risk of overt bleeding in the first 90 days of treatment, compared to genotype-predicted normal responders. Furthermore, there was minimal difference in bleeding risk between edoxoban and warfarin in those patients classified as normal responders to warfarin,^21^ indicating that preprescription genotyping may help stratify anticoagulant therapy.

Although there have been significant advances in the understanding of interindividual variability in response to warfarin, it is important to note that at least 40% of the variance in daily dose requirements remains unexplained, and is therefore a limitation of the reductionist approach. Other factors that may influence the response to warfarin such as microRNA^56^ and proteomics^57^ have been investigated, but further more global approaches that are integrated with the pharmacogenomic determinants are required.

##### Abacavir Hypersensitivity Syndrome

Up to 9% of patients that take abacavir can develop abacavir hypersensitivity syndrome (AHS)^58^; although the initial reaction is unpleasant, significant morbidity and mortality occurs particularly upon re-challenge,^59^ consistent with an immune-mediated delayed-type hypersensitivity ADR. In 2002, AHS was associated with HLA-B*57:01.^19^ The multicentre PREDICT-1 RCT demonstrated that pretherapy HLA-B*57:01 screening and avoidance of abacavir in patients carrying HLA-B*57:01 completely eliminated abacavir hypersensitivity reactions which had been immunologically confirmed by skin patch testing.^20^ This 100% negative predictive value was complemented with a positive predictive value of 47.9% and a number needed to screen to prevent one AHS case, given a 6% prevalence of HLA-B*57:01, of ∼25.^20^ As 84% of the PREDICT-1 participants were Caucasian, the retrospective case–control SHAPE study addressed this limitation and demonstrated that HLA-B*57:01 has 100% sensitivity for immunologically confirmed AHS in both US Black and White patients.^60^ The association between HLA-B*57:01 and the AHS represents the epitome of translational pharmacogenomics and widespread clinical implementation has occurred, improving abacavir's safety profile. Mechanistically, abacavir has been shown *in vitro* to bind noncovalently and specifically to HLA-B*57:01, altering its peptide-binding groove structure to create a HLA neo-allotype with a novel peptide binding portfolio^61^; the other system factors which determine whether or not abacavir binding to HLA-B*57:01 results in clinical AHS are less clear.

##### Tacrolimus Dose Requirements

The immunosuppressive drug, tacrolimus, shows high interindividual variability in its bioavailability, ranging from 5–90% with an average of ∼25%.^62^ Tacrolimus is a CYP3A substrate; CYP3A5 seems to play a more dominant role in tacrolimus metabolism than CYP3A4.^62^ The intronic *CYP3A5* SNP, rs776746 (6986A > G), that defines CYP3A5*3 is associated with alternative splicing, resulting in protein truncation and a substantial decrease in functional CYP3A5.^63^ Meta-analyses have reported that patients expressing CYP3A5 (*1 carriers) require significantly higher mean daily tacrolimus doses compared to CYP3A5 nonexpressors (*3/*3 patients) as determined by target tacrolimus therapeutic trough levels,^22^ and CYP3A5*3 may reduce acute rejection risk in the first month post transplantation.^22^ In a notable pharmacogenomic RCT involving 280 renal transplant recipients, prospective *CYP3A5* genotype-based tacrolimus dosing compared to the standard daily regimen significantly increased the proportion of patients reaching a therapeutic trough concentration (by day 3 of tacrolimus) and reduced dose modifications.^64^ However, there was no association between *CYP3A5* genotype and clinical endpoints, including acute rejection.^64^ This may relate to the small sample size, the use of therapeutic drug monitoring, concomitant medications (e.g., the use of high dose mycophenolate mofetil in all patients) and/or the incompletely understood relationship between tacrolimus PK and ADRs.^62^

CYP3A4*22 (rs35599367, 15389C > T) is associated with decreased human liver CYP3A4 mRNA. In renal transplant recipients, CYP3A4*22 carriage has been associated with increased tacrolimus dose-adjusted trough blood concentrations and reduced mean daily tacrolimus dose requirements to reach target levels independent of *CYP3A5* status, compared to homozygous *CYP3A4* wild-type patients.^65^,^23^ Both CYP3A5*3 and CYP3A4*22 SNPs considered together explain >60% of observed dose-adjusted tacrolimus trough blood levels.^65^ Beyond CYP3A5*3/CYP3A4*22, both POR*28 (rs1057868, A503V) within P450 oxidoreductase, which is the obligate electron donor for microsomal (including xenobiotic-metabolizing) CYPs, and SNPs in the nuclear receptor (NR) peroxisome proliferator-activated receptor α (*PPARA*), have also been associated with altered tacrolimus PK,^24^,^28^ albeit inconsistently. Collectively, although pharmacogenomics has accounted for a proportion of interindividual tacrolimus PK variability, there is limited understanding at present of the mechanisms underlying the differential susceptibility to tacrolimus ADRs (at similar whole blood concentrations) and so tacrolimus PK pharmacogenomics alone is unlikely to improve patient outcomes.^62^

Overall, even for the growing list of pharmacogenomic variants for prescribed drugs that are directly associated with ADRs, although they often have clinically acceptable negative predictive values, their clinical utility is severely limited because their typical positive predictive values range between just <1% to ∼20%.^66^ This occurs because risk allele frequencies are higher than the prevalence of their associated ADR. Therefore, the integration of other system factors alongside pharmacogenomics is required to improve risk prediction accuracy.

#### Epigenomic Factors of Drug Response Variability

Classically, epigenomics represents the study of gene expression alterations that are heritable between somatic cell mitotic divisions but are not attributable to the DNA sequence. Prototypical epigenomic mechanisms are DNA methylation and post-translational histone modifications, although regulation by noncoding RNAs, including microRNAs, are increasingly classed as epigenomic phenomena. The epigenome is dynamic and influenced by many factors including the genome (e.g., DNA-methylation related SNPs), disease status, pharmacotherapy (e.g., valproic acid and hydralazine), and smoking (see later). There is also interest in the role of *in utero* and early life experiences influencing later life and epigenomics may be integral to such processes. For example, individuals exposed to war-time famine in 1944–45 had different methylation profiles in loci implicated in growth and metabolic disease (e.g., within the cholesterol efflux regulatory transporter, *ABCA1*) compared to same-sex unexposed siblings, in whole blood samples taken six decades after the famine.^67^ Therefore although epigenomics is reversible, previous life experiences likely influence drug response.

Several genes relevant to drug response, including genes encoding DMEs, XTs, NRs, and drug targets are under epigenetic control. For example, CYP3A4 exhibits high interindividual variation in hepatic expression and the 5′ region upstream of *CYP3A4* has highly variable CpG methylation sites in adult livers.^29^ Importantly, the methylation status of single CpG positions near the proximal promoter is associated with *CYP3A4* expression.^29^ Furthermore, many cancers exhibit varied and aberrant epigenetic profiles, affecting chemotherapy response. For example, hypermethylation of the gene encoding transcription factor AP-2 epsilon (*TFAP2E*) in colorectal cancers, compared to hypomethylation, is significantly associated with clinical nonresponse to 5-fluorouracil (5-FU)-based chemotherapy regimens.^30^ A recent genome-wide methylation study using DNA tumour samples from a randomized phase III clinical study investigating the effect of adjuvant chemotherapy (procarbazine, CCNU, and vincristine [PCV]) in patients with anaplastic oligodendroglial tumours or oligoastrocytomas showed that predicted *MGMT* (encoding *O*^6^-methylguanine DNA methyltransferase) promoter methylation was associated with increased overall survival with PCV, whereas those with unmethlyated *MGMT* promoter status did not benefit from PCV.^31^

MicroRNAs are noncoding RNAs, 18–25 nucleotides long,^68^ that lead to post-transcriptional mRNA regulation. Of the >2500 mature human miRNAs currently identified, the expression levels of a growing number are becoming associated with differential chemotherapy response, including miR-21, miR-141, and miR-205.^68^ Recently, miR-519a has been identified as a novel breast cancer oncomir, which co-suppresses several tumour suppressor genes, is associated with *in vitro* resistance to tamoxifen-induced apoptosis and higher miR-519a expression has been associated with poorer disease-free survival in oestrogen receptor positive breast cancer patients.^32^

#### Proteomic Factors of Drug Response Variability

Proteomics is the large-scale study of proteins, particularly their expression, functions, and structures, and is shaped by interactions between genomic, environmental, and other biological levels. The quantity of protein available for interaction with a drug or other biological molecules is determined principally by its rates of synthesis, protein processing, and degradation and these influence drug response. For example, during inflammation, down-regulation of many CYPs and XTs occurs and one mechanism is cytokine-mediated CYP/XT transcriptional suppression to reduce their protein synthesis.^33^

There is growing interest in the complex proteome phenotype, particularly in oncology, to understand drug effects and stratify chemotherapeutic regimes. For example, quantitative proteomics has been applied to characterize drug response and detect potential therapeutic escape mechanisms in melanoma cell lines treated with novel inhibitors to heat shock protein 90 (HSP90) and mitogen-activated protein kinase kinase (MEK).^69^ Clinically, a multivariate serum protein test that assesses the intensity of eight serum mass spectra regions has been developed that can stratify patients according to whether they are likely to have a good or poor outcome with epidermal growth factor receptor (EGFR) tyrosine-kinase inhibitors, such as erlotinib. In a recent RCT of patients with nonsmall cell lung cancer randomized to either erlotinib or conventional chemotherapy, a pretherapy proteomic test classification of poor was associated with worse overall survival on erlotinib compared to chemotherapy, whereas there was no survival difference by treatment in patients with a proteomic test classification of good.^34^

#### Tissue and Organ Level Factors of Drug Response Variability

Both anatomical and (patho)physiological factors at the tissue/organ levels perturb drug response. Anatomically, tissue size variation affects parenchymal cell numbers and blood volume. For example for a given dose, increased adiposity increases the absolute amount of lipophilic drug distribution into fatty tissue. Both anatomically smaller kidneys and livers (e.g., in infants) and pathologically reduced nephron (e.g., in chronic kidney disease) and hepatocyte (e.g., in cirrhosis) abundance decreases absolute drug metabolism and excretion, with the net effect of increasing drug residence time.

Physiologically, interindividual variability in intestinal transit time is predicted to affect fractional absorption of poorly soluble, slow release, and/or low permeability drugs.^36^ Interestingly, after total gastrectomy, AUC and *C*_max_ of 5-FU following ingestion of the S-1 composite (composed of 5-FU, tegafur, and two 5-FU modulatory compounds) was significantly higher than presurgery,^38^ indicating increased 5-FU bioavailability. It has also been observed that hepatic microvasculature damage and hepatic blood congestion precede hepatocellular injury in paracetamol hepatotoxicity. This suggests that altered blood flow is involved in the pathogenesis, although its exact role remains unknown.^37^

### Environmental Factors

Exposure to exogenous substances including concomitant drugs, food, alcohol, and tobacco modify drug response. For example, environment-specific factors can perturb PD through drug–drug interactions (DDIs), which can have additive, synergistic or antagonistic effects. Classically, concurrent verapamil and beta blocker administration is known to predispose to severe bradycardia and hypotension, due to excessive inotropic and chronotropic effects.

Secondly, environmental factors modify PK. For example, the fractional absorption of levothyroxine in the gut lumen is reduced through chelation by concurrent iron, calcium or chromium supplementation or sevelamer administration.^39^ Concurrent food intake (especially fatty food) reduces gastric-emptying and slows intestinal drug absorption. Additionally, food may decrease the extent of absorption of drugs degraded in the stomach (e.g., penicillin G) and increase absorption of poorly soluble drugs (e.g., griseofulvin).^70^

Inhibition and induction of DMEs or XTs, through food- and DDIs, constitute major environmental mechanisms of target-drug PK modification. For example, grapefruit juice inhibits intestinal CYP3A4 and the antiretroviral combination of ritonavir and saquinavir is associated with a several fold increase in simvastatin acid AUC and *C*_max,_^40^ attributable to inhibition of both the hepatocyte-specific sinusoidal XT, OATP1B1,^41^ and CYP3A4,^40^ which are required for hepatic uptake and intestinal/hepatic metabolism of simvastatin acid, respectively.

DME and transporter expression is regulated by a complex interplay of gene regulatory pathways influenced by NRs, and NRs can mediate drug- and food-induced induction. For example, rifampicin binds the pregnane X receptor (PXR) leading to heterodimerization with retinoic acid receptor and induction of phase I and II DMEs and transporters including CYP3A4, uridine diphosphate glucuronosyltransferase 1A1 and P-glycoprotein, respectively.^42^ Another example is activated vitamin D (originating from food, supplementation, or UV-mediated synthesis), which binds the vitamin D receptor (VDR) and is a strong intestinal *CYP3A4* inducer. Interestingly, significant seasonal variation in duodenal CYP3A4 mRNA alongside an inversely related trend in midazolam AUC has been reported, likely reflecting seasonal variation in UV sunlight exposure.^45^ Interestingly, *CYP1A1* and to a lesser extent *CYP1A2* are induced by tobacco smoking and smoking is associated with reduced pulmonary *CYP1A1* promoter region methylation indicating smoking-associated epigenomic modifications.^43^ Furthermore, the AUC following single-dose erlotinib is significantly lower in smokers compared to nonsmokers, and although erlotinib is primarily metabolized by CYP3A4, both CYP1A1 and CYP1A2 also contribute.^71^ Finally, the antiepileptic drug carbamazepine causes CYP3A4 autoinduction through inhibition of histone deacetylase 1 binding to the CYP3A4 promoter, independent of PXR, suggesting carbamazepine-induced epigenomic perturbation.^44^

## SYSTEMS PHARMACOLOGY FOR PARSING INTERINDIVIDUAL DRUG VARIABILITY

Given the range and complex interplay between factors that modify drug action, conventional approaches are usually unlikely to describe the variation adequately to achieve clinical translation. Systems pharmacology holds potential for a deeper parsing of interindividual drug response variability, through harnessing both rapidly advancing drug-centred systems biology and pharmacometrics in an integrated and iterative manner. In doing so, systems pharmacology offers a holistic approach for further identification, characterization and quantitative integration of factors associated with drug perturbation.

### Pharmacologically-orientated Systems Biology

Pharmacologically-orientated systems approaches are developing along four main interconnected fronts: collation of increasingly large well-characterized patient samples, implementation of novel omics technologies, amassment of Big Data from multiple sources into publically available databases in conjunction with network-based analysis, and development toward structural systems biology and pharmacology. Firstly collaborations, such as the international Serious Adverse Event Consortium (iSAEC), alongside phenotype standardization initiatives,^72^ are facilitating international patient recruitment to increase sample sizes and collect blood samples for biobank storage whilst concomitantly ensuring high quality phenotypic data is captured using consistent definitions to reduce intra- and inter-study heterogeneity. For ADRs that occur along a spectrum, such as carbamazepine drug-induced skin injury and statin-induced myotoxicity, phenotype standardization is especially important.

Secondly, besides traditional GWAS, novel omics technologies and bioinformatics methods are being increasingly implemented in clinical pharmacology studies. For example, next-generation sequencing facilitates investigation of rare as well as common variation. In a study of drug-associated torsades de pointes, 23% Caucasian patients carried a highly conserved rare nonsynonymous variant compared to 1.7% of population controls (p = 0.0027) within 22 congenital arrhythmia genes.^73^ High-throughput omics technologies are increasingly being used to measure the dynamic intermediate biological levels to further characterize drug exposure phenotype and facilitate identification of novel drug response associations. For example, pharmacometabolomics identified higher postaspirin levels of inosine and adenosine in individuals classified as poor responders to aspirin compared to good responders, determined by *ex vivo* aspirin-induced platelet reactivity, and pharmacometabolomics-informed pharmacogenomics identified rs16931294 (14820A > G) in adenosine kinase to be strongly associated with differential aspirin response.^35^ Similarly, metabolomics determined that elevated pretreatment plasma glycine levels may be a risk marker for decreased response to escitalopram in patients with major depressive disorder; subsequent genotyping identified rs10975641 in glycine dehydrogenase to be associated with treatment outcome phenotypes.^74^ Multi-omics *in vitro* approaches also facilitate novel insight into ADR pathogenicity. For example, single and integrative transcriptomic, proteomic and metabolomics analyses of mouse hepatocytes, following exposure to the prototypical hepatotoxicant cyclosporin A, revealed mechanisms underlying cyclosporin A cholestasis including endoplasmic reticulum stress.^75^

Thirdly, a transition into an era of Big Data is rapidly occurring with data storage in large, often publically available online repositories. These data are being amassed from multiple sources including the increasing use of omics technologies to analyze patient-derived biological samples, systematic cell line transcriptomic profiling after genetic or pharmacological perturbation, systematic investigations of biological molecule interactions (e.g., gene-regulatory and protein–protein interactions) and increasing drug-centric data (e.g., phenotype and physiochemical data). Table 2 provides examples of publically available databases relevant to studying drug variability. Network description and analysis has emerged as a powerful tool to intelligently combine, visualize, and interrogate heterogeneous Big Data.^9^ One function of network analysis is prediction of new drug targets. For example, construction of a single node type drug–drug relation network based on phenotypic side effect similarity of 746 marketed drugs uncovered 261 unexpected drug-drug relations formed of chemically dissimilar drugs from different therapeutic indications that share a common side effect.^76^ Complementary *in vitro* experimentation confirmed binding activity to at least one predicted target for 13 of 20 tested unexpected but network-predicted drug pairs. For example, the proton pump inhibitor rabeprazole was newly confirmed to bind the DRD3 and HTR1D receptors, which are known targets of pergolide.^76^

**TABLE 2:** Key Publically Available Data Resources that Can Be Utilized and Integrated for Systems Pharmacology Analyses

Resource	Description	URL
Biological General Repository for Interaction Datasets (BioGRID)	Genetic and protein interaction data for different species including humans	http://thebiogrid.org/
Cancer Target Discovery and Development (CTD^2^)	Cell line fitness following genetic or drug perturbation, and data from judicious animal model testing	https://ocg.cancer.gov/programs/ctd2
ChEMBL	Database of small molecule bioactivities	https://www.ebi.ac.uk/chembl/
Connectivity Map (CMAP) and Library of Integrated Network-based Cellular Signatures (LINCS L1000)	Human mostly cancer cell line gene expression signatures following drug or endogenous ligand perturbation	http://lincs.hms.harvard.edu/explore_/canvasbrowser/
DrugBank	Drug (chemical, pharmacological and pharmaceutical) and drug target information	http://www.drugbank.ca/
Encyclopedia of DNA Elements (ENCODE)	Genomic map of gene regulatory elements, including transcription factor and histone modification binding sites	https://www.encodeproject.org/
US Food and Drug Administration (FDA) Adverse Event Reporting System (FAERS)	Adverse events and medication errors submitted to the FDA	http://www.fda.gov/Drugs/GuidanceComplianceRegulatoryInformation/Surveillance/AdverseDrugEffects/
Gene Expression Omnibus (GEO)	Gene expression signatures from cell lines and tissues following genetic or drug perturbation	http://www.ncbi.nlm.nih.gov/geo/
Gene Ontology (GO)	Species-independent functional annotation of gene products by associated biological processes, cellular components and molecular functions	http://geneontology.org/
Genomics of Drug Sensitivity in Cancer	Fitness of multiple cancer cell lines to drug perturbation, correlated to cell line genomic and expression data	http://www.cancerrxgene.org/
Genotype-Tissue Expression Project (GTEx)	Expression quantitative trait loci (eQTL), derived from expression signatures of multiple human tissues	http://www.gtexportal.org/home/
Interactome3D	Protein-protein interaction network with structural annotations	http://interactome3d.irbbarcelona.org/
International Mouse Phenotype Consortium (IMPC)	Systematic determination of gene knockout-mouse phenotype associations	https://www.mousephenotype.org/
Kyoto Encyclopedia of Genes and Genomes (KEGG)	Biological molecular interaction pathways/systems, genomic, chemical and drug related information	http://www.genome.jp/kegg/
The miRNA Pharmacogenomics Database (PharmacomiR)	Literature-derived miRNA pharmacogenomic data	http://www.pharmaco-mir.org/
Online Mendelian Inheritance in Man (OMIM)	Compendium of all known mendelian disorders and multifactorial diseases with a genetic component, focusing on genotype-phenotype associations	http://www.omim.org/
The Pharmacogenomics Knowledgebase (PharmGKB)	Clinical drug information, gene-drug and genotype-phenotype associations	https://www.pharmgkb.org/
Protein Data Bank (PDB)	Three-dimensional structural information of large biological molecules, predominantly proteins, from multiple species	http://www.rcsb.org/pdb/home/home.do
Roadmap Epigenomics	Development toward reference epigenomes for a range of human cells	http://www.roadmapepigenomics.org/
Side Effect Resource (SIDER)	Recorded ADRs of marketed drugs	http://sideeffects.embl.de/
Therapeutic Targets Database (TTD)	Established and exploratory drug target data, corresponding drug data and links to associated targeted pathways and diseases	http://bidd.nus.edu.sg/group/cjttd/

Distinguishing real signals from noise within empirical (especially omics) data using conventional statistics is challenging. However, network-based analyses can filter empirically derived data to gain nonintuitive insight, uncover novel associations and prioritize further research by defining the biological context of targets involved in therapeutic and adverse actions.^77^ One method is creating a seed list from existing knowledge and using it as an input for network-building computational algorithms. For example, recently, 167 rhabdomyolysis-inducing drugs (RIDs) served as the seed list for construction of a bipartite pharmacological network with edges to 272 known protein targets.^78^ The drug–protein target interaction data was sourced from DrugBank, Therapeutic Targets Database, and PharmGKB databases.^78^ This network was extended through inclusion of ‘intermediate’ proteins that interact with any of the known drug targets by either protein-protein or genetic interactions according to the Biological General Repository for Interaction Datasets (BioGRID) database. Subsequent enrichment analysis identified 78 novel intermediate proteins significantly associated with the rhabdomyolysis network compared to random drug sets.^78^ However, the target space of existing drug–target and protein–protein interaction databases is incomplete. Therefore, a complementary analysis of Connectivity Map empirical gene expression drug perturbation data, for 75 RIDs where this data was available, was undertaken. Of the 9899 genes whose expression was altered by at least 1 RID, *CPT2* (carnitine palmitoyltransferase II) was in the top 1% of most commonly perturbed genes by this group of drugs.^78^ *CPT2* mutations have previously been associated with lipid lowering therapy-associated myopathy,^79^ but this finding highlights its potential importance, prioritizing it for further study.

As generic *in silico*-based networks are not tissue-specific, a recent cardiac-specific long QT syndrome (LQTS) proteomics network was developed experimentally via immunoprecipitation of five known LQTS proteins from cardiac mouse tissue; the proteomics network was constructed from these five seed proteins and interacting proteins identified in the precipitates.^80^ This network was integrated with results from a large recent GWAS that had associated common variant loci with QT interval duration. Importantly, after excluding the congenital LQTS proteins, 12 proteins from the network were encoded by genes within these loci, prioritizing candidate genes for further functional assessment to elucidate causal mechanisms underlying these unexplained loci. Secondly, the network was used to filter SNPs modestly associated (p < 10^−3^) with QT duration according to whether the SNPs were located near genes of network proteins. Selected SNPs were genotyped in a replication cohort and three reached genome-wide significance when meta-analyzed with the initial GWAS: rs10824026 (*VCL*), rs889807 (*SRL*), and rs7498491 (*TUFM/EIF3C/EIF3CL*).^80^ As QT-prolongation is frequently multifactorial, these common variants plausibly contribute to an individual's risk of drug-induced Torsades de Pointes. Therefore collectively, these examples illustrate the potential of network-based analysis to integrate with, filter and augment complex empirical data. Furthermore, such networks (consisting of seed and interacting nodes associated with clinical drug response phenotypes) are neither overly simplistic nor prohibitively complex. Importantly, this compromise makes network construction and analysis a rational means of defining the suitable ‘molecular space’ for targeted in-depth biochemical kinetics analyses, facilitating quantitative pharmacometric modelling.^77^

Lastly, it is increasingly recognized that a physiochemical molecular level understanding of protein–protein interactions and protein–drug interactions is essential for a deeper understanding of interindividual differences in system responses to drug perturbation. To illustrate, approximately 20% of clinically apparent drug resistance to imatinib develops through acquisition of the T315I Abl gatekeeper mutation in the imatinib oncoprotein on-target, Bcr-Abl, and the substitution from wild-type threonine to isoleucine sterically blocks imatinib binding. This structural insight is facilitating development of new Bcr-Abl inhibitors, such as ponatinib, which are capable of inhibiting T315I Bcr-Abl.^15^ Furthermore ligand-induced, specific protein conformational states can result in selective signalling (biased agonism), which is thought to modulate, for example, the downstream signalling selectivity of G-protein coupled receptors, a major drug-target class.^81^ Therefore, structural systems biology is developing from the systematic integration of structural data into biological networks (e.g., networks derived from experimentally identified protein–protein interactions), which when integrated with pharmacometric modelling approaches, is leading to the emergence of ‘structural systems pharmacology’. For example, antibacterial mechanisms of compounds in *Escherichia coli* K12 have been predicted using a structural-based algorithm to predict antibacterial protein targets from a genome-scale model of metabolism integrated with protein structures (GEM-PRO) and expanded to incorporate multimeric metabolic enzyme structures.^82^ Although a comprehensive interactome-wide structurally annotated resource does not yet exist, recent breakthroughs in membrane protein crystallography and the rapidly increasing number of protein-protein, protein-ligand and protein-nucleic acid three-dimensional complexes being deposited in the Protein Data Bank (PDB) are advancing structural systems pharmacology,^83^ with potential for advancing our understanding of structure-based differential drug response.

### Pharmacometrics

**FIGURE 3:**
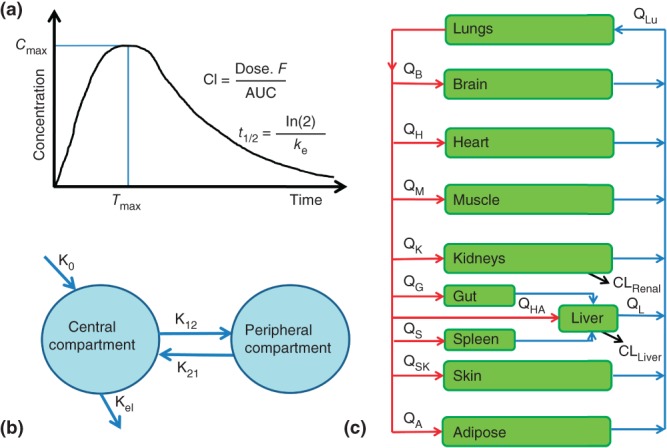
Overview of the main pharmacokinetic modelling methods. (a) Noncompartmental analysis is the preferred method to determine overall drug exposure (i.e., AUC), using the trapezoidal rule, and other pharmacokinetic parameters (e.g., *C*_max_, clearance, elimination half-life, etc.) as it involves few assumptions.^84^ (b) Compartmental and (c) physiologically based pharmacokinetic models (PBPK) are constructed from compartments that are interconnected using differential equations that describe drug flow between model constituents. Conventional compartmental models are constructed from one or more compartments that are descriptive, rather than mechanistically representative; the final model is parsimonious and compartments are only included if they noticeably improve the final model fit to the empirical data. PBPK models include multiple compartments that represent actual physiology (i.e., organs and blood), incorporate data from more diverse sources, and if properly validated can be used to make PK predictions and extrapolations for circumstances (e.g., different doses or routes of administration) beyond those used to construct the model.^85^ (Reprinted with permission from Ref. 86; Copyright 2013.

The intrinsic quantitative properties of pharmacology advocate that quantitative integration of factors associated with variable drug response is the ideal. PK models predominate and can be subdivided into three major types of increasing complexity: noncompartmental, compartmental, and physiologically based PK (PBPK) models (Figure 3).^86^ Briefly, noncompartmental analysis is useful for determining overall drug exposure (i.e., AUC), using the trapezoidal rule, and other PK parameters (e.g., *C*_max_, clearance, etc.).^84^ Compartmental models are based on one or more descriptive, nonphysical compartments, whereas PBPK models include multiple biologically representative compartments; both are constructed using differential equations that describe rates of change in drug between model constituents. Similarly, an increasingly sophisticated range of PD models are emerging from basic models to enhanced PD (ePD) and small PD systems models.^86^ Basic PD models include simple direct effect models, based on the Hill equation, and indirect response, signal transduction, and irreversible effect models constructed from small numbers of differential equations; in contrast ePD/small PD systems models are composed from multiple differential equations. PK models are frequently integrated with basic PD models to generate mechanistic PK/PD models, and theoretically PK models could be combined with ePD and systems models to generate enhanced physiologic and PK/PD systems pharmacology multiscale models, although to the best of the authors' knowledge such steps have yet to be published. Traditional noncompartmental/compartmental PK and basic PD models are ‘top–down’ models, tightly fitted to experimental data, seek parsimony and model parameters are expected to have strong statistical reliability. However, the increasingly complex PBPK and PD models are ‘bottom up’ constructions less robustly data-driven, utilize diverse data sources to assign parameters when possible (including omics data), estimate parameters when necessary and are principally used for simulation and exploration.^86^

Population PK (POP PK) modelling is an example of a rigorous data-driven PK modelling approach, integral to model-based drug development and can identify and confirm candidate factors associated with interindividual drug plasma concentration variability. For example, a compartmental POP PK model of human plasma simvastatin/simvastatin acid levels was recently constructed from three studies and three clinical factors plus seven SNPs in the genes encoding the XTs OATP1B1, ABCG2 and OATP2B1, the DMEs CYP3A4 and CYP3A5 and the NR PPARA were identified as covariates that significantly affect model parameters.^25^ rs4149056 (V174A) in *SLCO1B1* (encoding OATP1B1) is an established risk factor for simvastatin-induced myopathy^26^; therefore it will be interesting to determine whether, in addition to rs4149056, the other identified pharmacogenomic PK variants collectively modify the risk of this ADR. However, like all human studies, the ability of POP PK to detect factors is limited by their effect size, prevalence, the sample size, and access to pharmacologically relevant tissues (e.g., blood, gut, and liver). Nevertheless, POP PK modelling with incorporation of ADME pharmacogenomic variants is a useful approach and can run alongside the development of new drugs through integration of the early (rich PK) and late (sparse PK) phase clinical trial data. However, for drugs already in clinical practice, either interlinked rich/sparse PK *de novo* studies or collaboration with industry and genotyping of samples from old PK studies are required, and these represent barriers to the more general uptake of this methodology.

PBPK models are organ level systems models and, although not a new concept, a rapid expansion of PBPK utilization has occurred over the last few years attributable to several reasons including increased computing power and the greater connectivity between *in vitro–in vivo* extrapolation (IVIVE) and PBPK modelling.^87^ The extrapolation of *in vivo* intrinsic organ clearance from *in vitro* systems, such as recombinantly expressed enzymes, human liver microsomes or hepatocytes, using appropriate scaling factors, can facilitate the quantitative modelling of human system factors such as genetically perturbed DME function.^87^ Although rigorously data-driven models (e.g., POP PK) are more reliable, PBPK-IVIVE simulations help rationally prioritize the most appropriate factors for targeted empirical investigation, predict PK profiles of patient groups with altered physiology (e.g., infants and children^86^) and provide a framework for construction of models to integrate multiple factors associated with interindividual PK variability, enabling investigations into their variously combined quantitative effects.^87^ Although empirical knowledge of the interindividual variable size of scaling factors is increasing, further investigations (e.g., quantitative proteomics) are required to refine the scaling up of *in vitro* data into PBPK-IVIVE models.^87^

PBPK(−IVIVE) modelling can be combined with basic PD models to integrate the three potential rate-limiting steps of PK, drug–target interaction and turnover processes reflecting physiological homeostasis or disease mechanisms.^86^ For example, the integration of *in vitro* kinetics data for rosuvastatin and the XTs OATP1B1, OATP1B3, OATP2B1, NTCP, and ABCG2, with clinical data and a modified indirect response model resulted in a whole-body PBPK-IVIVE/PD model that innovatively used predicted hepatic unbound intracellular concentration of rosuvastatin for the PD input, rather than total plasma rosuvastatin concentration. Importantly, the known association between large increases in rosuvastatin plasma concentration in the presence of rs4149056, the deleterious SNP in OATP1B1, and just minor reductions in cholesterol lowering efficacy, could be modelled.^27^

A greater understanding of molecular pathways downstream of drug targets has led to the concept of ePD models. For example, simulations using an ePD model formed from 34 nonlinear ordinary differential equations predicted the effect of EGFR inhibition (e.g., by gefitinib) on tumour growth after adjusting for the effects of DNA methylation status to the *RASAL1* promoter, a coding SNP in *RKIP/PEBP* and altered miR-221 expression, which all differentially perturb downstream EGFR signalling.^88^ Drug-induced inhibition of 80% of EGFR activity was assumed. Interestingly, although tumour size response varied between simulated patients depending on parameter combinations, it could be classed into three main treatment outcome groups, illustrating the potential for pharmacometrics to facilitate precision medicine.^88^

Similarly, complex modelling of multiple molecular interactions generated a quantitative multiscale systems model of integrated calcium homeostasis and bone remodelling. This model was extended using RCT data on the antiresorptive agent denosumab to facilitate the prediction of nonlinear longitudinal changes in the clinical surrogate endpoint of lumbar spine bone mineral density during and following discontinuation and reinstitution of denosumab.^89^ In general, although few such systems pharmacology models yet exist, they should facilitate systematic simulation-driven predictions of the effect of differing combinations of factors on drug response within a systems context. Targeted empirical corroboration may validate and iteratively refine models and provide a route for translating model-derived predictions into clinical drug stratification.

## CONCLUSION

Although RCT evidence has unequivocally demonstrated the overall clinical benefit of multiple marketed drugs, interindividual variability in drug response creates inequality in the benefit derived. For a considerable proportion of patients, a given drug may be clinically ineffective or harmful carrying deleterious repercussions for the patient's health and the efficient use of limited healthcare resources. Pharmacogenomics continues to advance our understanding of the effects of genetic variation on drug response, although the lack of consistency between studies and inadequate predictive values is hampering widespread clinical uptake. Notwithstanding the necessity of continual methodological improvements, the limited translational success of pharmacogenomics may reflect its one-dimensional approach to the complexities of the multiscale network-based human body system and its interactions with drugs; therefore it is probable that more complex answers are required to adequately parse drug variability. As well as enabling new target identification for drug development, the interdisciplinary field of systems pharmacology holds significant potential for identifying and characterizing the constituents of variability and their interconnections in an integrated systems context to provide a deeper understanding of the mechanisms underlying interindividual drug variability. Several barriers exist including, the intelligent and easy integration of the rapidly accruing Big Data that spans different biological domains and databases, the complexities of multiscale modelling, the determination of optimal model detail to sufficiently parse drug variability without being cumbersome and universally acceptable evidential standards to permit clinical translation from this novel field. Nevertheless, systems pharmacology is rapidly developing to meet these challenges and help realize the goal of precision medicine, although much work remains.
